# Evaluation of Online Consumer Health Information for Idiopathic Scoliosis Identified by a Google^TM^ Search

**Published:** 2018-11-29

**Authors:** Sarah C. Heady, Marissa A. Weaver, Gina M. Berg, Emily M. Manlove, Jennifer E. Thuener, Douglas C. Burton

**Affiliations:** 1 Baylor University, Waco, TX; 2 University of Kansas School of Medicine-Wichita, Department of Family and Community Medicine; 3 University of Kansas School of Medicine-Kansas City, Department of Orthopedic Surgery

**Keywords:** scoliosis, eHealth, consumer health information, internet, patient education

## Abstract

**Introduction:**

This study sought to assess the quality of online consumer health information about idiopathic scoliosis. Previous studies showed that quality of online health information varies and often lacks adherence to expert recommendations and guidelines. Nevertheless, 72% of internet users seek health information online. A 2005 analysis of online scoliosis information found that the information was limited and of poor quality.

**Methods:**

Two reviewers vetted the top 10 websites resulting from a GoogleTM search for “scoliosis.” Content was organized into categories and rated by three physician evaluators using a 1 – 5 scale based on quality, accuracy, completeness of information, readability, and willingness to recommend. Additional information, such as number of ads and Flesch-Kinkaid reading level, also was collected.

**Results:**

The average overall physician score was 47.6 (75 possible). All websites included content that was mostly accurate but varied in completeness. Physicians unanimously recommended Mayo Clinic, MedicineNet, and Kids Health; none recommended the Google^TM^ Knowledge Graph. The Scoliosis Research Society website reached the highest overall physician score. Readability ranged from 7^th^ grade to college level; only that of Kids Health was below 10^th^ grade level.

**Conclusions:**

Most essential information provided by the websites was accurate and generally well rated by physicians. Website ranking by physicians was inconsistent with the ranking order by Google^TM^, indicating that health seekers reviewing the top Google^TM^-ranked websites may not be viewing the websites rated highest by physicians. Physicians should consider patient literacy in website recommendations, as many have an above average literacy level.

## INTRODUCTION

Idiopathic scoliosis (IS), which accounts for 80% of scoliosis in adolescents, is a “three-dimensional torsional deformity of the spine and trunk” of unknown origin.^1^ It is divided into three main classifications by age of onset: infantile, juvenile, and adolescent. Approximately 90% of IS cases develop in adolescent patients between 11 – 18 years of age. Overall, adolescent idiopathic scoliosis (AIS) is estimated to affect 0.47 – 5.2% of the global population.^2^

For decades, scoliosis screenings were a routine part of school physical examinations of adolescents.^3^ In 2004, the U.S. Preventive Services Task Force (USPSTF) published a recommendation against routine screening, concluding that screening does not increase probability of early diagnosis significantly due to the variable accuracy of the forward bending test and poor follow-up of patients diagnosed in screening.^4^ Furthermore, USPSTF concluded that potential detriments of screening (unnecessary referral, radiation exposure, and bracing) outweighed the benefits of potential earlier diagnosis.

Several physician organizations, including Society on Scoliosis Orthopaedic and Rehabilitation Treatment (SOSORT),^1^ Scoliosis Research Society, Pediatric Orthopedic Society of North America, American Academy of Orthopedic Surgeons, and American Academy of Pediatrics, hold that additional research on scoliosis screening since the 2004 USPSTF recommendation has documented benefits of earlier detection and non-surgical treatment of IS.^5^ An updated 2018 USPSTF recommendation concluded that there is currently insufficient evidence to weigh the harms and benefits of adolescent idiopathic scoliosis screening.^6^

Evident lack of consensus in the scientific community on scoliosis screening and lack of conclusive scientific evidence on the effectiveness of conservative treatments (like observation, physical therapy, and bracing) and surgical treatments confer great import to patient preferences. Therefore, SOSORT recommends that patient/caretaker education, psychotherapy, assessment of patient co-operation, support groups, and internet forums be available to help patients and caretakers navigate the scoliosis treatment process.^1^

The internet can be a valuable source of information on scoliosis, particularly considering the ambiguity of screening recommendations and complexity of IS treatments. Those concerned with scoliosis may use online information to self-screen or look for treatment options. A 2013 Pew Research Center survey reported that 87% of American adults use the internet and 72% had searched online for health information within the past year. Most (77%) used a general search engine like Google^TM^ or Bing^TM^.^7^ Online search information influences how consumers manage their care and may serve as a substitute for seeking treatment from a medical professional.^8^,^9^ However, online information often lacks peer review and must be scrutinized carefully.^8^,^10^

Regarding internet-informed self-diagnosing, 41% reported that their self-diagnosis was confirmed by a medical professional and 18% reported medical professional disagreement, while 35% did not visit a clinician for confirmation.^9^ Studies evaluating internet health information for such topics as concussions^11^,^12^, child safety education^13^, nutrition^14^, breast cancer^15^–^19^, inflammatory bowel diseases^20^–^23^, acute low back pain^24^–^26^, and eye conditions^27^ have shown such information to be lacking in adherence to expert recommendations and guidelines. In fact, an earlier analysis of online scoliosis information (evaluated 2003, published 2005) found that the information was limited and of poor quality and concluded that physicians must assume primary responsibility for patient education.^28^

Patients searching for health information online face the obstacle of high literacy requirements. The vast majority of online sources have reading levels that are inappropriate for the general U.S. population.^29^–^32^ Daraz et al.^29^ showed mean reading levels between 10^th^ to 15^th^ grades of nearly 8,000 websites, depending on the scale used to measure reading level. According to the 2003 national assessment of adult reading levels by the National Center for Education Statistics, the average reading level of a typical American is between 7^th^ to 8^th^ grade level. The U.S. Department of Health and Human Services (USDHHS) developed a scale that qualifies material under a 6^th^ grade reading level as “easy to read;” between 7^th^ to 9^th^ grade level as “average difficulty,” and above 9^th^ grade level as “difficult.” Most online health sources have a “difficult” reading level on this scale.^32^ According to the USDHHS, limited health literacy is correlated with poorer health outcomes.^33^ Thus, it is important that online information on scoliosis has a reading level accessible to most of the U.S. population.

Overall, patient education is important for patients with scoliosis due to the controversy over screening and treatment. Patients and their caretakers need ready access to accurate health information at an appropriate reading level, and most patients rely on the internet to supply such health information. However, internet health information on scoliosis has not been evaluated systematically for several years. Thus, this study aimed to evaluate online scoliosis information for accuracy and readability.

## METHODS

In June 2016, websites containing information about IS were identified using the keyword “scoliosis” on the search engine Google^TM^. Google^TM^ scored the highest in search validity in a comparative study on the quality of search engines for online medical information.^34^ The top 10 ranked non-media-related scoliosis websites were evaluated^35^ because the average online health consumer views only the first few websites and rarely goes beyond the first page of results.^36^,^37^

Each website was vetted individually by two investigators (SH and MW) and organized into categories. “Essential information” was defined via information typically required by patient informed consent forms^38^ and included definition, types of scoliosis described, demographics of those affected, causes and risk factors, signs and symptoms, screening and diagnosis, types of curves, treatment options, self-management tips, and complications of untreated scoliosis. “Additional information” included myths about scoliosis, current research, surgery recommendations, chiropractic cures, and extra information. Non-evaluated descriptive information also was collected: recommendations for speaking with a physician and routine scoliosis screening, Flesch-Kincaid reading grade levels, the number of front-page advertisements, availability of patient handouts and privacy policies, and the presence of research article citations. The researchers merged their individual content assessments and resolved any content discrepancies through discussion.

Website content information was blinded for source (host and universal resource locator [URL]) evaluation by copying and pasting website material into standardized forms. This content was reviewed by three physicians: two family medicine physicians and an orthopedic spinal deformity specialist. The physicians were asked to review and evaluate the categorical information and score the information on a scale of one (1 = Poor) to five (5 = Excellent) based on quality, accuracy, and completeness of information. Categories listed on the physician evaluation forms as containing “no information” provided by the website were assigned scores of zero (0) for consistency. There were two missing physician scores (out of 450 possible); these were replaced with the average of the other two physician scores for that section. Physicians’ scores were averaged by category and the total essential (50 possible) and overall (75 possible) scores for each website were calculated. The “essential” score was calculated from the score totals in the “essential categories,” as listed above. The “overall” score was calculated from the score totals in all the categories, including “essential” and “additional” ones, as listed above. Physician rankings were created using the website essential scores and the overall (essential plus additional category) scores. The physicians also rated their “willingness to recommend this website to patient consumers” between “strongly disagree”, “disagree”, “agree”, or “strongly agree”. These ratings were dichotomized to agree/disagree for analysis. Three-way interrater agreement was assessed by using intraclass correlation coefficient^39^ per SPSS for Windows Version 23.^40^ Interpretation was based on the classification suggested by Landis and Koch.^41^ Correlation between the Google^TM^ ranking and the physician ranking was analyzed using the Spearman rank order correlation coefficient.

## RESULTS

Table 1 lists the web addresses for all ten websites evaluated in Google^TM^-ranked order. Tables 2 and 3 contain categorical evaluations of the top ten Google^TM^-ranked websites, including the Google^TM^ Knowledge Graph sidebar. The mean physician evaluation scores for each category are found in Table 2, along with the essential and overall physician scores and rankings. Table 3 contains the website ratings for the “additional information” categories and descriptive information about the websites.

The overall average physician scores were 47.63/75, with 6/10 websites scoring 50 or above. Scoliosis Research Society scored the highest with 62.67/75 and Google^TM^ Knowledge Graph scored the lowest with 15.67/70. When the outlier score of 15.67 for the Google^TM^ Knowledge Graph was dropped, the average score increases to 51.19. The top three rankings by method included: WebMD, Mayo Clinic, and Medicine Net as ranked by Google^TM^; Medicine Net, Spine Health, and Wikipedia as ranked by essential information scores; and Scoliosis Research Society, Spine Health, and WebMD as ranked by the overall physician scores. Medical News Today, Kids Health, and the Google^TM^ Knowledge Graph ranked consistently in the 8^th^, 9^th^, and 10^th^ spots respectively, regardless of ranking method. The Spearman correlation test resulted in a significant correlation between physicians’ ranking of essential information and overall scores (p < 0.005) and not significant between physicians’ ranking of overall scores and the Google^TM^ ranking (p = 0.187). The physicians unanimously agreed to recommend only three (Mayo Clinic, Medicine Net, Kids Health) of the ten websites evaluated; none were willing to recommend Google^TM^ Knowledge Graph. Overall, the correlation coefficient was 0.308 (CI: −1.107, 0.816), indicating interrater agreement was fair.^41^

Table 2 lists the individual scores for each category of content on each website. It also includes the total scores for the essential categories and overall categories on each website. Regarding essential information, the average physician score total was 36.03/50. Medicine Net scored the highest with 47/50 and Google^TM^ Knowledge Graph scored the lowest with 15.67/50. When the outlier score of 15.67 for the Google^TM^ Knowledge Graph was dropped, the average essential score increases to 38.30. All (10/10) websites had information but considerable variations in score ranges in the categories describing scoliosis (2.33 – 4.67), demographics (1.33 – 5.0), sign and symptoms (2.67 – 4.67), screening/diagnosis (3.0 – 5.0), and treatment information (2.0 – 5.0). Half (5/10) of the websites were missing identifiable information describing complications. The information on the Google^TM^ Knowledge Graph was sparse (9/15 categories contained no information, 4 in essential [types, causation/risk, curves, and complications]) and consistently received low scores.

Only 2/3 physicians said they would recommend each of the top two overall ranked websites to patients; however, 3/3 physicians would recommend the 9^th^ ranked website, Kids Health.

Over half (6/10) of the websites were missing information on current research; half (5/10) also were missing information regarding myth debunking, surgery above a given Cobb angle, and chiropractic cures. Google^TM^ Knowledge Graph did not include any of the additional information evaluated.

Most websites (8/10, including Google^TM^ Knowledge Graph) recommended speaking with a physician and almost all (9/10, not including Google^TM^ Knowledge Graph) recommended routine screening. Half (5/10) provided patient handouts and half (5/10) cited research. Half (5/10) had advertisements on their front pages with the number of ads ranging from 2 – 5. Most (9/10) listed privacy policies (not including Scoliosis Research Society). Reading grade levels ranged from 7^th^ grade to college; only Kids Health had a reading grade level below 10^th^ grade.

## DISCUSSION

This study sought to evaluate accuracy and readability of scoliosis information available on the top ten Google^TM^-ranked websites. Most websites provided accurate, but not complete, information. Most provided “essential information,” though explanations about curves, self-management tips, and information about complications more often were neglected. “Additional information” was absent on many websites, though available information generally was rated well by the physicians. Such information may not be critical for decision-making about treatment options but may be helpful for patients.

The evolution of internet information on scoliosis is evident in comparing this study with an earlier evaluation of online scoliosis information published by Mathur et al. in 2005.^28^ In the Mathur study, 50 websites from five search engines (MSN, Yahoo, AltaVista, Google, and Lycos) were considered. Currently, most searches are powered by Google™ (64.0%) and Bing (21.4%).^42^ Six of the 10 websites assessed in this study had predecessors assessed in the Mathur study. In both the Mathur study and this study, srs.org, the official website of the Scoliosis Research Society, was ranked #1 for accuracy and completeness by physician reviewers. Notable among the four websites not included in the Mathur study are Wikipedia (now the 5^th^ most visited website with over 4.75 million articles, but in 2003 relatively new with just over 100,000 articles^43^) and the Google™ Knowledge Graph, a new addition by Google™ as of 2015.^44^ In the Mathur study^28^, 90% of websites surveyed scored under 50% in content quality (completeness), and 36/50 websites scored 50% or less in accuracy. Our study showed that 7/10 websites surveyed scored over 50% in average overall physician score (quality and completeness), and 8/10 websites surveyed would be approved for content quality, accuracy, and readability by at least 2/3 of physician examiners. The quality of readily available online information on scoliosis appeared to increase considerably in the 13-year time span between the content evaluation of these two studies.

The Google^TM^ Knowledge Graph, a relatively new feature released in 2015 to provide relevant medical information on specific conditions, indicates the continuing need to evaluate content of online information. The official Google^TM^ blog reports that a team of physicians from Google^TM^ and/or Mayo Clinic compiled the information, but includes the disclaimer that the search results are not intended as medical advice.^44^ This feature appears as a sidebar on a standard computer screen or at the top of a mobile Google^TM^ search. It contains three categories of information, “About,” “Symptoms,” and “Treatments,” with brief information on the relevant condition. However, it consistently ranked 10^th^ on both the overall and essential physician scores lists (the graph was not given a Google^TM^ ranking since it takes the form of a sidebar). This evaluation indicates that the highly visible Knowledge Graph was not complete in the information provided for scoliosis. Further research is necessary to evaluate the quality of information provided by the Google^TM^ Knowledge Graph feature.

Regarding readability, this study seems to confirm that the high reading level of health information online remains a significant concern. Five websites were found to be written at a 10^th^ or 11^th^ grade level; four at a college level; and one at a college graduate level. The average reading level of American adults is 7^th^ or 8^th^ grade level,^32^ but only the Kids Health website scored within those grades per the Flesch-Kinkaid reading level assessment. Information should be at a level of completeness, accuracy, and readability suitable for an average non-medically-trained patient.

A serendipitous finding of this study was the incongruence of physician recommendation patterns with the overall physician rankings of the websites. Although the spine specialist almost always scored the quality and completeness of information lower than the family practitioners did, the specialist only disagreed with recommending two of the websites to patients, while both family practitioners disagreed with recommending four (not all the same). Evaluation notes left by the physicians indicated that the family physicians were concerned with the complexity and readability of the information. This may be due to differing purposes of website recommendation. Family practitioners may consider ease of use and readability of information more often because they are introducing patients to scoliosis. Spine specialists work with patients that have been diagnosed already and may be seeking more detailed information, particularly treatment information, online.

This study may be limited by the number of evaluating physicians (three) and variance on responses. This study was limited to the use of Google^TM^ and did not include other search tools, such as Bing^TM^, which may result in different search result rankings. Another limitation is that the physician evaluation of the websites was not randomized; systematic bias may have been introduced, as they all were reviewed in the same order as Google^TM^ rank. Also, two opportunities for scoring were overlooked by the evaluating physicians and replaced with the mean of the other two evaluators; this may contribute to unrepresentative scores. Finally, only 10 websites were evaluated (per reported consumer behavior^36^,^37^); an assessment of a larger number of scoliosis websites might provide a more complete perspective on online scoliosis information reliability.

Recommended future research should assess the accuracy and completeness of the Google^TM^ Knowledge Graph information for various common conditions, per its stated purpose, since it is so visible under search results. This is critical because consumers often rely on information immediately available on the web.^37^ An assessment of how patients respond to and use scoliosis information would inform more relevant website design and content. Furthermore, evaluating the priorities of differing specialties for recommendation of health information websites would be worthwhile, as this study noted that there were considerable differences. Finally, content on scoliosis and other healthcare websites should be evaluated regularly to inform practitioners as to the quality of information their patients may be using for decision-making.

## CONCLUSION

Healthcare consumers with scoliosis concerns likely will use the internet to seek information regarding scoliosis for better understanding and treatment decision making.^37^ Our study showed that most of the top ten websites found when searching for the term “scoliosis” using Google^TM^ have relatively accurate and complete information, but did have variation. Patients should seek for information from multiple sources to get complete information. Furthermore, patients should not rely on the ranking order given by Google^TM^, as the Google^TM^ rankings were not aligned with the physician scored rankings (overall or essential).

For recommendations on websites concerning scoliosis, practitioners should consider the needs of their patient population. Physicians, especially in primary care, should account for literacy of patients. Specialists may need to encourage patients to read websites with higher reading levels because detailed information becomes increasingly important for patients seeking specialist care.

## Figures and Tables

**Table 1:** Listing of top 10 Google^TM^ websites and URLs from search “scoliosis”.

Website	URL Used for Evaluation
WebMD	http://www.webmd.com/osteoarthritis/guide/arthritis-scoliosis
Mayo Clinic	http://www.mayoclinic.org/diseases-conditions/scoliosis/home/ovc-20193685
Medicine Net	http://www.medicinenet.com/scoliosis/article.htm
Niams.nih.gov	http://www.niams.nih.gov/health_info/scoliosis/
Spine Health	http://www.spine-health.com/conditions/scoliosis/scoliosis-what-you-need-know
Wikipedia	https://en.wikipedia.org/wiki/Scoliosis
Scoliosis Research Society	http://www.srs.org/
Medical News Today	http://www.medicalnewstoday.com/articles/190940.php
Kids Health	http://kidshealth.org/en/kids/scolio.html
Google^TM^ Knowledge Graph	N/A

**Table 2:** Physician overall and essential information scores of top 10 hits using “scoliosis” as search term in Google^TM^.

		Website	Scoliosis Research Society	Spine Health	Medicine Net	Mayo Clinic	Wikipedia	Niams.nih. gov	WebMD	Medical News Today	Kids Health	Google
	**Rank**	**By overall physician scores**	**1**	**2**	**3**	**4**	**5**	**6**	**7**	**8**	**9**	**10**
		**By essential physician scores**	**4**	**2**	**1**	**5**	**3**	**6**	**7**	**8**	**9**	**10**
		**Google^TM^**	**7**	**5**	**3**	**2**	**6**	**4**	**1**	**8**	**9**	**Knowledge Graph**
**Physician Scores (Average)**	**Overall Information**	Physician would recommend	2/3 Agree	2/3 Agree	3/3 Agree	3/3 Agree	1/3 Agree	2/3 Agree	2/3 Agree	2/3 Agree	3/3 Agree	0/3 Agree
		Average overall physician score (out of 75)	62.67	57.33	56.00	55.67	54.67	53.67	49.00	36.33	35.33	15.67
	**Essential Information**	What is scoliosis?	4.00	4.33	4.67	2.33	4.00	3.67	4.67	4.67	4.00	3.67
		Types of scoliosis	4.67	5.00	5.00	3.00	4.00	4.33	4.67	4.33	3.67	0
		Demographics of scoliosis	3.67	4.33	5.00	1.67	4.00	3.33	4.00	3.00	3.33	1.33
		Causes/risk factors	4.67	4.33	4.67	3.33	4.33	4.33	4.33	3.33	3.67	0
		Signs & symptoms	4.67	4.33	4.67	4.33	4.00	4.00	4.33	4.33	4.33	2.67
		Screening/diagnosis	4.33	4.67	4.67	5.00	4.00	4.67	3.67	4.33	4.33	3.00
		Types of curves	4.33	5.00	4.33	4.00	4.33	4.00	2.67	3.00	0	0
		Treatment information	4.67	5.00	5.00	5.00	4.33	4.67	4.33	3.67	4.67	2.00
		Self-management tips	4.67	4.67	4.67	5.00	4.00	4.67	3.67	0	0	3.00
		Complications	0	3.33	4.33	4.33	4.67	0	0	2.50	0	0
		Total physician essential score (out of 50)	39.67	45.00	47.00	38.00	40.33	37.67	36.67	32.33	28.00	15.67

**Table 3:** Physician evaluation of additional information and recommendations of top 10 hits using “scoliosis” as search term in Google^TM^.

		Website	Scoliosis Research Society	Spine Health	Medicine Net	Mayo Clinic	Wikipedia	Niams.nih.gov	WebMD	Medical News Today	Kids Health	Google
	**Additional Information**	Debunking myths	5.00	4.67	0	4.33	0	4.67	4.33	0	0	0
		Current research	4.33	0	0	4.33	4.67	4.67	0	0	0	0
		Surgery above Cobb Angle	4.67	0	4.33	0	5.00	0	4.00	0	3.00	0
		Chiropractic cures	4.67	2.67	0	4.67	0	3.50	4.33	0	0	0
		Extra information	4.33	5.00	4.67	4.33	4.67	4.33	0	4.00	4.33	0
**Descriptive Information**	**Recommend**	Speak with physician	Yes	Yes	Yes	Yes	No	Yes	Yes	No	Yes	Yes
		Routine scoliosis screening	Yes	Yes	Yes	Yes	Yes	Yes	Yes	Yes	Yes	No
	**Information**	Patient handouts	No	No	No	No	No	Yes	No	No	Yes	Yes
		Privacy policy	No	Yes	Yes	Yes	Yes	Yes	Yes	Yes	Yes	Yes
		Citations present	Yes	Yes	No	No	Yes	No	Yes	No	No	No
		# Front page ads	0	0	3	2	0	0	5	4	0	0
	**Reading Level**	Grade	College	College	10th	11th	College grad +	10th	11th	College	7th	10th
